# Next-Generation *EGFR* Tyrosine Kinase Inhibitors for Treating *EGFR*-Mutant Lung Cancer beyond First Line

**DOI:** 10.3389/fmed.2016.00076

**Published:** 2017-01-18

**Authors:** Ivana Sullivan, David Planchard

**Affiliations:** ^1^Department Medical Oncology, Gustave Roussy, Villejuif, France

**Keywords:** *EGFR*, T790M, NSCLC, osimertinib, third generation, brain metastasis

## Abstract

Tyrosine kinase inhibitors (TKIs) against the human epidermal growth factor receptor (*EGFR*) are now standard treatment in the clinic for patients with advanced *EGFR* mutant non-small-cell lung cancer (NSCLC). First-generation *EGFR* TKIs, binding competitively and reversibly to the ATP-binding site of the *EGFR* tyrosine kinase domain, have resulted in a significant improvement in outcome for NSCLC patients with activating *EGFR* mutations (L858R and Del19). However, after a median duration of response of ~12 months, all patients develop tumor resistance, and in over half of these patients this is due to the emergence of the *EGFR* T790M resistance mutation. The second-generation *EGFR/HER* TKIs were developed to treat resistant disease, targeting not only T790M but *EGFR*-activating mutations and wild-type *EGFR*. Although they exhibited promising anti-T790M activity in the laboratory, their clinical activity among T790M+ NSCLC was poor mainly because of dose-limiting toxicity due to simultaneous inhibition of wild-type *EGFR*. The third-generation *EGFR* TKIs selectively and irreversibly target *EGFR* T790M and activating *EGFR* mutations, showing promising efficacy in NSCLC resistant to the first- and second-generation *EGFR* TKIs. They also appear to have lower incidences of toxicity due to the limited inhibitory effect on wild-type *EGFR*. Currently, the first-generation gefitinib and erlotinib and second-generation afatinib have been approved for first-line treatment of metastatic NSCLC with activating *EGFR* mutations. Among the third-generation *EGFR* TKIs, osimertinib is today the only drug approved by the Food and Drug Administration and the European Medicines Agency to treat metastatic *EGFR* T790M NSCLC patients who have progressed on or after *EGFR* TKI therapy. In this review, we summarize the available post-progression therapies including third-generation *EGFR* inhibitors and combination treatment strategies for treating patients with NSCLC harboring *EGFR* mutations and address the known mechanisms of resistance.

## Introduction

Over the past decade, scientific advances have progressively improved outcomes for patients diagnosed with lung cancers driven by target oncogene mutations. The first oncogenic driver in non-small-cell lung cancer (NSCLC) was discovered in 2004 with the identification of activating mutations in the kinase domain of the epidermal growth factor receptor (*EGFR*) among patients with dramatic responses to *EGFR* tyrosine kinase inhibitors (TKIs) (1–3). *EGFR* mutations account for 10–17% of NSCLC cases in North America and Europe and 30–50% of NSCLCs in Asian countries and are most common among patients with adenocarcinoma NSCLC and a light or non-smoking history (4, 5). The first-generation TKIs gefitinib (Iressa^®^, AstraZeneca, London, UK) and erlotinib (Tarceva^®^, F. Hoffmann-La Roche, Basel, Switzerland), and the second-generation TKI afatinib (Giotrif^®^, Boehringer Ingelheim, Ingelheim, Germany) have shown higher response rates (RRs), improving progression-free survival (PFS) and quality of life compared to standard platinum-based chemotherapy in patients with good performance status (0–2) whose tumors harbor an activating (sensitizing) *EGFR* mutation (6–13).

These data established *EGFR* TKIs as the treatment of choice for patients with newly diagnosed *EGFR*-mutant advanced NSCLC. Of note, none of these studies demonstrated a benefit in terms of overall survival (OS) due to the high level of crossover. However, an unplanned pooled OS analysis of patients included in the LUX-Lung 3 or LUX-Lung 6 phase III trials demonstrated an OS benefit for afatinib compared to platinum-based chemotherapy in patients whose tumors harbor *EGFR* Del19 mutations vs. *EGFR* L858R mutations: 27.3 vs. 24.3 months, respectively [hazard ratio (HR) 0.81; 95% confidence interval (CI), 0.66–0.99; *p* = 0.037] (14). However, this benefit was not confirmed in the phase IIb LUX-Lung 7 designed to compare head-to-head afatinib with gefitinib in the first-line treatment of patients with *EGFR*-mutant NSCLC (15). Unfortunately, the majority of patients progress after a median of 12 months treatment with first-line TKIs, and multiple mechanisms of acquired resistance have been identified. Among them, the most common mechanism (~50% of cases) is the acquisition of a missense mutation within exon 20 of *EGFR*, the T790M mutation (p.Thr790Met) (16).

Until recently, standard chemotherapies were the main treatment option in a post-progression setting. For patients initiating chemotherapy, the role of *EGFR* TKI maintenance remains controversial. In a retrospective analysis, up to 23% of patients experience a disease flare after TKI discontinuation (17), which led many clinicians to continue *EGFR* TKIs when starting chemotherapy. It was hypothesized that some clones within a resistant cancer remained sensitive to *EGFR* inhibition and that withdrawal of the TKI could “let loose” these clones with resultant adverse outcomes. The randomized phase III IMPRESS trial provided the first prospective data to address this clinical question. Patients progressing on first-line gefitinib were randomized to receive cisplatin–pemetrexed with gefitinib or placebo. The trial did not confirm a benefit of maintaining the *EGFR* TKI, with comparable RRs and PFS in the two arms (18). The final OS analysis was presented recently; patients in the gefitinib arm had significantly lower OS compared to the placebo arm (13.4 vs. 19.5 months, HR = 1.44, *p* = 0.016), confirming the deleterious effect of maintaining the *EGFR* inhibition. Of note, this detrimental effect was predominantly observed among patients whose tumors harbored a T790M mutation detected *via* circulating tumor DNA (ctDNA; HR = 1.49; 95% CI, 1.02–2.21) (19).

To date, many third-generation *EGFR* TKIs have been developed to target both sensitizing *EGFR* mutations and *EGFR* T790M. In this review, we outline available post-progression therapies including osimertinib (previously known as AZD9291) as the only drug approved by the US Food and Drug Administration (FDA) and the European Medicines Agency (EMA) for the treatment of patients with metastatic *EGFR* T790M+ NSCLC who have progressed on or after *EGFR* TKI therapy (20), and other next-generation irreversible *EGFR* TKIs in clinical development (Table 1).

**Table 1:** ***EGFR* TKI generations for metastatic *EGFR*-mutant NSCLC**.

Generation TKI	Drug	Company	*EGFR* inhibition	Molecular targets	Most common adverse events	Status
First generation	Gefitinib	AstraZeneca	Competitive; reversible	*EGFR* L858R, Del19	Diarrhea, rash/acne, ALT/AST increased, decreased appetite	Phase III (approved)

	Erlotinib	F. Hoffmann-La Roche				

Second generation	Afatinib	Boehringer Ingelheim	Covalent; irreversible	wt-*EGFR, EGFR* L858R, L858R/T790M, L858R/T854A, wt-*HER2, HER2* amp., *HER4*	Skin rash, diarrhea	Phase III (approved)

	Dacomitinib	Pfizer		*EGFR* L858R, Del19, T790M, wt-*HER2*, mutant-*HER2, HER2* amp., *HER4*	Diarrhea, rash/acne	Phase III

	Neratinib	Puma Biotechnology		*EGFR* L858R, T790M, *HER2, HER4*	Diarrhea, dyspnea, nausea, vomiting	Phase III

Third generation	Osimertinib	AstraZeneca	Covalent; irreversible	*EGFR* L858R, Del19, T790M (limited activity against wt-*EGFR*)	Diarrhea, rash, nausea, decreased appetite	Phase III (approved)
	Rociletinib	Clovis			Hyperglycemia, long QT interval, nausea, fatigue, diarrhea	Phase II/III (stopped)
	Olmutinib	Hanmi/Boehringer Ingelheim			Diarrhea, rash, skin exfoliation, nausea, pruritus	Approved in South Korea^a^
	ASP8273	Astellas			Diarrhea, nausea, vomiting, platelet count decreased	Phase III
	Nazartinib	Novartis			Rash, diarrhea, pruritus	Phase I/II
	PF-06747775	Pfizer			No reported yet	Phase I/II
	Avitinib	Ace Bio			No reported yet	Phase I
	HS-10296	Jiangsu Hansoh			No reported yet	Phase I/II

*^a^Due to an unexpected increase of grade 3/4 skin toxicity, the ELUXA clinical trial program was temporally stopped*.

*ALT, alanine aminotransferase; AST, aspartate aminotransferase; Del, deletion; amp, amplification; TKIs, tyrosine kinase inhibitors; wt, wild-type; EGFR, epidermal growth factor receptor*.

## Identifying the Acquired Resistance Mechanism to First/Second-Generation *EGFR* TKIs

For patients whose disease progresses on gefitinib, erlotinib, or afatinib, understanding the major mechanisms of resistance is essential to choosing the optimal post-progression treatment. To date, repeated biopsies are the standard of care; however, this approach comes with some limitations—not all patients are amenable to this procedure, and not all progressing lesions are accessible for biopsy. In addition, there is growing evidence that a single biopsy may not accurately represent the intrinsic heterogeneity of a resistant tumor. Liquid biopsy is a valid alternative to tissue rebiopsy. This approach, which has been validated (21), represents a surrogate DNA source and is a novel strategy for tumor genotyping, mainly applicable at the time of progression for *EGFR*-mutated patients (22–24). In cases when the T790M mutation is identified in peripheral blood, treatment with third-generation *EGFR* TKIs is justified (25).

In addition to T790M, other resistance mechanisms have also been identified. Globally, these can be categorized as target gene alterations (i.e., *EGFR* amplifications and mutations such as T790M), downstream bypass signaling pathway activation (i.e., *MET* and *HER2* amplifications or mutations in *BRAF, PIK3CA*), and phenotypic changes (including small-cell lung cancer transformation and epithelial to mesenchymal transition) (26, 27).

## Targeting *EGFR* T790M+ NSCLC

### Second-Generation *EGFR* TKIs

Following the discovery that T790M is the main resistance mechanism against the first-generation *EGFR* TKIs gefitinib and erlotinib, many new drugs targeting T790M were developed. Although second-generation *EGFR* inhibitors such as neratinib, afatinib, and dacomitinib exhibited promising anti-T790M activity in the laboratory, their clinical activity in T790M+ NSCLC was poor, with RR less than 10% among patients resistant to gefitinib or erlotinib (28–30). In addition, increased toxicity, mainly skin and digestive (Table 1), was observed due to *EGFR* wild-type inhibition at lower concentrations than those required to inhibit T790M. Thus to date, none of the second-generation agents are considered as effective monotherapies in patients progressing on first-generation TKIs.

On the basis of preclinical observations that afatinib plus cetuximab (an anti-*EGFR* monoclonal antibody) overcame T790M-mediated resistance (31), this combination was evaluated in a phase Ib trial enrolling 126 heavily pretreated patients with advanced *EGFR*-mutant NSCLC who had developed resistance to erlotinib/gefitinib. The overall response rate (ORR) was 29% and was comparable in both T790M+ and T790M− tumors (32 vs. 25%), and median PFS was 4.7 months (95% CI, 4.3–6.4) (32). However, the dual *EGFR* inhibition resulted in increased toxicity with various grades 3–4 adverse events (AEs) (mainly rash, diarrhea, and fatigue) reported in up to 46% of patients (32). A randomized phase II/III trial (NCT02438722) of afatinib plus cetuximab vs. afatinib alone is currently open in treatment-naïve patients with advanced *EGFR*-mutant NSCLC.

### Third-Generation *EGFR* TKIs

Many third-generation *EGFR* inhibitors are currently being consecutively developed to more effectively target the T790M mutation. Unlike second-generation TKIs, as these drugs exhibit increased specificity for T790M and thus mutant *EGFR* compared to wild-type *EGFR*, they are well tolerated resulting in few wild-type *EGFR* adverse effects. Among them, osimertinib (AZD9291) was the first to receive FDA and EMA approval in November 2015 and February 2016, respectively, for metastatic *EGFR* T790M+ NSCLC, which has progressed on or after *EGFR* TKI therapy. Table 2 shows available efficacy data of new-generation *EGFR* TKIs.

**Table 2:** **Efficacy of third-generation tyrosine kinase inhibitors (TKIs) in activating epidermal growth factor receptor (*EGFR*) mutations and T790M+ NSCLC patients**.

	Osimertinib				Rociletinib	Olmutinib	ASP8273	Nazartinib
Trial	AURA phase I	AURA phase I T790M+	AURA phase II ext.	AURA2 phase II	TIGER-X phase I/II^a^	HM-EMSI-101 phase I/II T790M+ (ongoing)	NCT02113813 phase I/II (ongoing)	NCT02108964 phase I/II (ongoing)
							
			T790M+					
			Pooled analysis					

Patients (*N*)	253 T790M+ = 138	63	201	210	69 T790M+ = 51	76	63 T790M+ = 58	152

Dose	20–240 mg qd	80 mg qd	80 mg qd		500, 625, or 750 mg bid	800 mg qd	300 mg qd	75–350 mg qd

ORR act*EGFR*m (%)	51 [95% CI, 45–58]	–	–		17 [95% CI, 4–41]	–	30	–

ORR T790M+ (%)	61 [95% CI, 52–70]	71 [95% CI, 57–82]	66 [95% CI, 61–71]		45 (95% CI, 31–60)	62	29 (central testing)	46.9

Overall mPFS (95% CI) mo	T790M+: 9.6 (8.3–NR) T790M−: 2.8 (2.1–4.3)	9.7 (8.3–13.6)	11.0 (9.6–12.4)		T790M+: 6.1 (4.2–9.6) T790M−: 1.8 (1.2–3.0)	6.9 (5.4–9.5)^b^	T790M+: 6.8 (5.5–NR)^c^ T790M−: 6.0 (4.1–9.8)	9.7 (7.3–11.1)

Reference	Jänne et al. (36)	Yang et al. (37)			Sequist et al. (41)	Park et al. (44), Lee et al. (45)	Yu et al. (49)	Tan et al. (51)

*^a^Updated results from 69 reviewed cases included in the phase I TIGER-X trial*.

*^b^Updated results from 2016 ASCO Annual Meeting*.

*^c^mPFS from 28 NSCLC patients with central testing T790M+*.

*actEGFRm, activating EGFR mutation; bid, twice daily; CI, confidence interval; ext., extension; mo, months; mPFS, median progression-free survival; NR, not reached; ORR, overall response rate; qd, once daily*.

#### Osimertinib (AZD9291; Tagrisso^®^)

Osimertinib is a mono-anilino-pyrimidine compound that acts as a covalent *EGFR* TKI. In *EGFR* recombinant enzyme assays, osimertinib showed potent activity against diverse *EGFR* mutations (L858R, L858R/T790M, exon 19 deletion, and exon 19 deletion/T790M) and exhibited nearly 200 times greater potency against L858R/T790M than wild-type *EGFR*. Osimertinib is metabolized to produce at least two circulating metabolites, AZ5104 and AZ7550. In biochemical assays, AZ7550 had a comparable potency and selectivity profile to osimertinib, although AZ5104 showed greater potency against exon 19 deletions, T790M mutants (both ~8-fold) and wild-type (~15-fold) *EGFR* (33). Its pharmacokinetic exposure did not significantly differ between Asian and non-Asian patients, showing a minimal food effect (34). Additionally, data from a clinical pharmacokinetic study (NCT02163733) showed that osimertinib exposure was not affected by concurrent administration of omeprazole (35). Thus, unlike first- and second-generation TKIs, gastric pH modifying agents can be concomitantly used with osimertinib without restrictions.

A phase I/II dose-escalation study of osimertinib (AURA, NCT01802632) was carried out in patients with locally advanced or metastatic *EGFR*-mutated NSCLC progressing on first- or second-generation *EGFR* TKIs. Patients were not preselected according to T790M status (36). The study included 253 patients who received osimertinib at five dose levels ranging from 20 to 240 mg daily and distributed between two cohorts, dose-escalation and dose-expansion cohorts. Among 31 patients enrolled in the dose-escalation cohort, no dose-limiting toxicity (DLT) occurred and the maximum tolerated dose (MTD) was not reached. An additional 222 patients were treated in five dose-expansion cohorts. The *EGFR*-T790M mutation was detected in tumors from 138 patients (62%) in the expansion cohorts. Of the 253 patients treated across all dose levels, 239 were evaluated for response. The ORR and disease control rate (DCR) in the whole population were 51% [95% CI, 45–58%] and 84% [95% CI, 79–88%], respectively. Among the 138 patients with a centrally confirmed *EGFR*-T790M mutation, 127 patients were evaluable for response. Outcomes were substantially better in the *EGFR* T790M+ population compared to T790M− tumor patients with an ORR of 61% [95% CI, 52–70%] vs. 21% [95% CI, 12–34%], a DCR of 95% [95% CI, 90–98%] vs. 61% [95% CI, 47–73%] and median PFS of 9.6 months [95% CI, 8.3–not reached] vs. 2.8 months [95% CI, 2.1 to 4.3], respectively (36). There were no DLTs at any dose level. The most common AE, mostly grade 1–2, were diarrhea (47%), skin toxicity (rash/acne, 40%), nausea (22%), and anorexia (21%). With increased incidence and severity of AEs (rash, dry skin, and diarrhea) in relation to the wild-type *EGFR* inhibition at higher dose levels (160 and 240 mg), 80 mg daily was selected as the recommended dose for further clinical trials (36).

The efficacy and safety data from the 80 mg expansion cohort in patients with centrally confirmed T790M NSCLC were recently updated (data cutoff: January 4, 2016). Among 63 patients, 61 patients were evaluable for response. The ORR and DCR were 71% [95% CI, 57–82%] and 93% [95% CI, 84–98%], respectively, with a median PFS of 9.7 months [95% CI, 8.3–13.6] (37).

The 80-mg daily dose evaluated in the phase II T790M+ extension cohort of the AURA trial (described above) was evaluated in an additional phase II “AURA2” study (NCT02094261) designed for patients with confirmed *EGFR*-mutant T790M+ locally advanced or metastatic NSCLC progressing on an approved *EGFR* TKI. A preplanned pooled analysis of both studies was performed. Among 411 patients (201 from the AURA extension and 210 from AURA2), 397 were evaluable. The ORR and DCR were 66% [95% CI, 61–71%] and 91% [95% CI, 88–94%], respectively. Median PFS was 11.0 [95% CI, 9.6–12.4] months with a median duration of response of 12.5 months [95% CI, 11.1 months to not calculable] (37).

Osimertinib has also demonstrated activity in the first-line setting. Data from two expansion cohorts in treatment-naïve *EGFR*-mutated advanced NSCLC patients were recently presented. Sixty patients received osimertinib 80 mg (*n* = 30) or 160 mg (*n* = 30) once daily and all were evaluable. The confirmed ORR was 77% [95% CI, 64–87%] with a DCR of 98% [95% CI, 89–100%]. Median PFS was 19.3 months [95% CI, 13.7 to not calculable] (38).

A number of phase III trials involving osimertinib in different settings are ongoing. The phase III FLAURA trial (First-Line-AURA; NCT02296125) in *EGFR*-mutated treatment-naïve NSCLC patients was designed to compare osimertinib 80 mg daily vs. the current standard of care gefitinib or erlotinib. The AURA3 trial (NCT02151981) is an open-label, randomized trial in the second-line setting, designed to compare osimertinib with platinum-based doublet chemotherapy in patients with *EGFR* T790M+ locally advanced or metastatic NSCLC. In a press release dated July 18, 2016, AstraZeneca announced that the AURA3 trial, which included more than 400 patients, had met its primary endpoint demonstrating superior PFS compared to standard platinum-based chemotherapy.

In the adjuvant setting, the ongoing ADAURA trial (ADjuvant-AURA; NCT02511106) is a double-blind, randomized, placebo-controlled trial assessing the efficacy and safety of osimertinib vs. placebo in patients with *EGFR*-mutated stage IB–IIIA NSCLC following complete tumor resection. Results are not yet available.

#### Rociletinib (CO-1686)

Rociletinib is another oral, irreversible, mutant-selective inhibitor of commonly mutated forms of *EGFR*, including T790M, with minimal activity against wild-type *EGFR* in preclinical studies (39). A phase I/II trial (TIGER-X; NCT01526928) of rociletinib was performed in patients with *EGFR*-mutant NSCLC with acquired resistance to first- or second-generation *EGFR* TKIs (40). In the expansion (phase II) part of the study, patients with T790M+ NSCLC received rociletinib at doses of 500, 625, or 750 mg twice daily. At the time of report, 130 patients were enrolled. The MTD was not identified. The most common grade 3 AE was hyperglycemia, occurring in 20 of the 92 patients (22%) who received therapeutic doses. Among the 46 evaluable patients with T790M+, the ORR was 59% [95% CI, 45–73%]. For the 17 evaluable patients with T790M− disease, the ORR was 29% [95% CI, 8–51%] (40).

In November 2015, Clovis Oncology issued a press release that contained data from a pooled analysis of TIGER-X and TIGER-2 (NCT02147990), another phase II trial examining rociletinib in second line in patients with *EGFR* T790M+ NSCLC progressing on at least on *EGFR* inhibitor. Among 325 patients, the ORR (dose range, 500–750 mg twice daily) was 30.2% [95% CI, 25.2–35.5%]. The ORRs were 32% [95% CI, 25–40%] and 23% [95% CI, 14–34%] in patients receiving 625 mg (*n* = 170) and 500 mg (*n* = 79), respectively. The median duration of response for the two treatment doses was 8.8 and 9.1 months, respectively. Due to the different RR, an independent updated analysis was assessed in patients (intention-to-treat population) included in the TIGER-X trial confirming ORRs of 45% [95% CI, 31–60%] and 17% [95% CI, 4–41%] among patients with T790M+ and T790M− disease, respectively (41). Clovis thus decided to halt enrollment in all ongoing rociletinib studies, including the phase III TIGER-3 trial (NCT02322281), and has withdrawn its application for regulatory approval in the European Union.

#### Olmutinib (BI-1482694/HM61713; Olita™)

Olmutinib is an oral *EGFR* mutant-specific TKI active against mutant *EGFR* isoforms, including T790M, while sparing wild-type *EGFR* (42). A phase I/II trial HM-EMSI-101 (NCT01588145) was conducted to evaluate the safety, tolerability, pharmacokinetics, and preliminary activity of olmutinib in Korean patients with *EGFR* TKI-pretreated NSCLC (43). Patients received olmutinib at doses ranging from 75 to 1,200 mg/day. The ORR was 58.8% in the 34 patients who received olmutinib with a dose more than 650 mg. The most common DLTs involved gastrointestinal symptoms and increased aspartate aminotransferase, alanine aminotransferase, amylase, and lipase levels. The recommended phase II dose was 800 mg/day. In part II of the study, 76 patients with centrally confirmed T790M+ NSCLC were enrolled, 70 of whom were evaluable for response. The ORR was 61% and median PFS (*n* = 76) was 6.9 months [95% CI, 5.36–9.49]. The most common drug-related AEs (all grades) were diarrhea (59%), pruritus (42%), rash (41%), and nausea (39%) (44). These data validate previous preliminary trial results presented at the European Society for Medical Oncology Asia Congress in December 2015 (45). These results were the basis for Breakthrough Therapy Designation granted by the FDA in 2015 and the first approval for the treatment of patients with *EGFR* T790M+ NSCLC in South Korea in 2016. Following promising early clinical data, Boehringer Ingelheim launched the ELUXA clinical trial program to investigate olmutinib as a monotherapy in different settings as well as in combination with other anticancer treatments. Nevertheless, due to an unexpected increase in grade 3/4 skin toxicity (epidermolysis) in previous trials Boehringer decided to definitively stop the development of this drug.

#### ASP8273

ASP8273 is another oral, irreversible TKI that inhibits the kinase activity of *EGFR* mutations including T790M, with limited activity against *EGFR* wild-type (46). ASP8273 was further shown to suppress signaling *via* ERK and Akt. This agent showed activity in mutant *EGFR* cell lines that are resistant to other *EGFR* TKIs including osimertinib and rociletinib (47). ASP8273 was evaluated in an open-label phase I/II study (NCT02192697) for safety and efficacy (48). Thirty Japanese patients were enrolled in the phase I dose-escalation cohorts across seven dose levels (25–600 mg/day), and 15 patients were enrolled in the response expansion cohorts across four dose levels (100–400 mg/day). T790M status was 49% positive, 13% negative, and 38% unknown, respectively. Responses were observed in patients enrolled in ≥100 mg/day cohorts. Partial responses were achieved in 50% (18/36) of all evaluable patients and 80% (12/15) of patients with T790M+ NSCLC (including confirmed and unconfirmed). The most common AEs (all grades) were diarrhea (56%), nausea (31%), vomiting (31%), and thrombocytopenia (31%). Based on tolerability and preliminary antitumor activity, the recommended phase II dose selected was 300 mg once daily (48). The safety, tolerability, and antitumor activity for ASP8273 300 mg/day in patients with NSCLC *EGFR* mutation-positive and previously treated with an *EGFR* TKI were recently presented in a total of 63 patients, including seven treated in the dose-escalation part, 18 in the response expansion, 19 in recommended phase II dose part, and 19 from the food effect cohort (49). The majority of tumors (>90%) were positive for the T790M mutation based on local testing. All but one patient (98%) had been previously treated with an *EGFR* TKI, with erlotinib the most common inhibitor. Among the 63 patients treated with ASP8273 300 mg, the ORR was 30% [95% CI, 19.2–43.0%] and the median PFS was 6.0 [95% CI, 4.1–9.8] months. For the subgroups with T790M+ tumors the ORRs, assessed by local or central testing, were similar: 31% [95% CI, 19.5–44.5%] and 29% [95% CI, 13.2–48.7%], respectively. Median PFS for T790M+ patients (local testing) was 6.0 months [95% CI, 5.3–9.8] and 6.8 months [95% CI, 5.5 months to not evaluable] for T790M+ patients (central testing). The most frequent drug-related AEs (all grades) were diarrhea (48%), nausea (27%), hyponatremia (19%), paresthesia (14%), and vomiting (13%). Six patients (10%) discontinued treatment due to treatment-related toxicity (49). Based on this study, the dose of ASP8273 300 mg daily was selected for a recently initiated, large (*n* = 600), international, randomized, phase III study (SOLAR) to compare the clinical efficacy and safety/tolerability of ASP8273 with erlotinib or gefitinib as initial treatment of advanced *EGFR*-mutant NSCLC (NCT02588261).

#### Nazartinib (EGF816)

**Figure 1:**
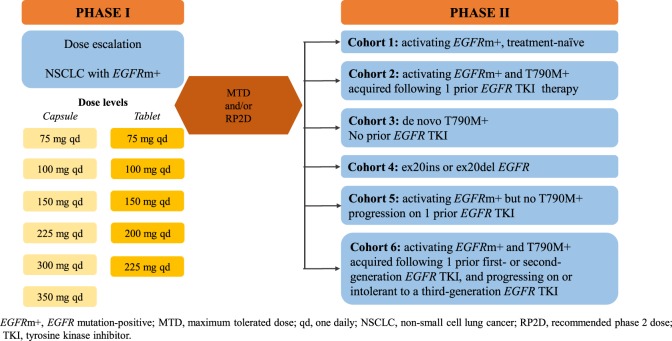
**Study design of nazartinib in *EGFR*m+ NSCLC patients (NCT0210896)**. *EGFR*m+, *EGFR* mutation-positive; MTD, maximum tolerated dose; qd, one daily; NSCLC, non-small-cell lung cancer; RP2D, recommended phase 2 dose.

Nazartinib is a novel, irreversible mutant-selective *EGFR* inhibitor that specifically targets both *EGFR*-activating mutations (L858R, Del19) and the resistant T790M mutation, while sparing wild-type *EGFR* (50). NCT02108964 (EGF816X2101) is a phase I/II first-in-human study of nazartinib in patients with *EGFR*-mutated locally advanced or metastatic NSCLC. Updated results from the phase I dose-escalation part were recently presented. Patients were assigned to receive once-daily nazartinib with doses ranging from 75 to 350 mg. At the cutoff date of January 29, 2016, 152 patients had been treated across seven cohorts (51). Among them, 147 patients were evaluable for response. The confirmed ORR was 46.9% [95% CI, 38.7–55.3%] and the DCR was 87.1% [95% CI, 80.6–92.0%]. The estimated median PFS across all dose levels was 9.7 months [95% CI, 7.3–11.1]. Among 69 patients with confirmed responses at the cutoff date, the estimated median duration of response was 9.5 months [95% CI, 9.2–14.7]. The most common toxicities (all grades) were rash (54%), diarrhea (37%), and pruritus (34%). Interestingly, the rashes observed in the study tended to have a different pattern, location, and histology than those seen with other *EGFR* TKIs that target wild-type *EGFR*. Diarrhea was the most common grade 3/4 AE (16%), and of note, both incidence of diarrhea and rash tended to increase with increasing nazartinib doses (51). The phase II part, performed in six cohorts, is ongoing (Figure 1). In addition, the drug is being investigated in association with INC280, a specific *MET* inhibitor (based on the potential escape pathway for third-generation *EGFR* TKIs) in an ongoing phase Ib/II trial in patients with advanced *EGFR* mutant NSCLC (NCT02335944), and with nivolumab, an anti-PD-1 monoclonal antibody in a phase II trial in *EGFR* mutant/T790M+ NSCLC patients who have progressed on first-line *EGFR* TKI (NCT02323126).

#### Avitinib (AC0010)

Avitinib is another new-generation inhibitor of *EGFR* that, like the abovementioned agents, targets *EGFR*-activating mutations overcoming T790M-induced mutation with limited activity against wild-type *EGFR*. Clinical trials were initiated in China and the United States in parallel using avitinib as second-line therapy in NSCLC patients progressing on first-generation EGFR TKIs and who have acquired the gatekeeper T790M mutation. Two trials evaluating the safety, tolerability, pharmacokinetics, and antitumor activity of avitinib are ongoing; a phase I/II trial (NCT02274337) in advanced NSCLC patients progressing on prior therapy with an *EGFR* TKI agent and a phase I trial (NCT02330367) designed to determine the MTD and/or recommended phase 2 dose in previously treated mutant *EGFR* NSCLC patients with a T790M resistant mutation.

#### PF-06747775

PF-06747775 is another small molecule inhibitor of *EGFR* T790M with minimal activity against wild-type *EGFR*. It is being studied in a phase I/II clinical trial (NCT02349633) in advanced NSCLC patients with *EGFR* mutations (Del19 or L858R ± T790M). Results are not yet available.

#### HS-10296

HS-10296 is a small molecule inhibitor of *EGFR*-activating mutations and T790M-resistant mutation with limited activity against wild-type *EGFR*. An open-label, multicenter, phase I/II trial of HS-10296 with dose escalation, dose expansion, and extension cohorts in locally advanced or metastatic NSCLC patients who have progressed following prior therapy with an *EGFR* TKI agent is currently recruiting participants (NCT02981108).

## Third-Generation *EGFR* TKIs in Central Nervous System (CNS) Metastases

Incidence data of brain and leptomeningeal metastasis in *EGFR*-mutated NSCLC patients come from retrospective cohorts, reporting 24 and 9%, respectively. Gefitinib, erlotinib, and afatinib have impressive intracranial activity, with RR of 60–80% (52, 53). However, for patients with CNS progression on these first- and second-generation agents, further effective therapies are limited. Among the third-generation *EGFR* TKIs, osimertinib was the only inhibitor demonstrating sustained tumor regression in both preclinical and clinical models (54). Osimertinib has greater penetration of the mouse blood–brain barrier than gefitinib, rociletinib, or afatinib, and induced sustained tumor regression in an *EGFR* mutant PC9 mouse brain metastasis model at clinically relevant doses, while rociletinib did not achieve tumor regression (55). CNS activity was confirmed in the AURA study phase II extension cohort (NCT01802632) and the AURA2 phase II study (NCT02094261) (56). The phase I BLOOM trial (NCT02228369) was designed to assess for the first time the safety, tolerability, pharmacokinetics, and preliminary antitumor activity of two third-generation *EGFR* TKIs, orsimetinib and AZD3759. AZD3759 was the first *EGFR* TKI primarily designed to effectively across the blood–brain barrier to tackle CNS metastases in patients with *EGFR* mutant NSCLC (57, 58). The osimertinib cohort of the trial included 21 Asian patients with advanced or metastatic NSCLC harboring the L858R mutation (*n* = 13) or an exon 19 deletion (*n* = 9), and a confirmed diagnosis of leptomeningeal metastasis by positive cerebrospinal fluid cytology. At study entry, the T790M mutation was detected in cerebrospinal fluid in two patients and in plasma in six. Patients received osimertinib at 160 mg/day. All patients were evaluable for efficacy; seven (33%) had a confirmed radiologic response, nine (43%) had stable disease, and neurological function improvement was seen in five (24%) patients (59). Preliminary results from the AZD3759 cohort were recently presented (60). Twenty-nine patients with advanced *EGFR* mutant NSCLC and brain metastases, including leptomeningeal metastasis were treated in escalating dose cohorts of 50–500 mg, twice daily (BID). The pharmacokinetic analysis demonstrated excellent CNS penetration, with a 1:1 ratio with plasma. The tolerability profile of AZD3759 was consistent with *EGFR* TKI class effects and included grade 3/4 rash (7%), pruritus (7%), diarrhea (3%), and acne (3%). The MTD was 300 mg BID but study investigators recommended 200 mg BID for phase II dosing. AZD3759 demonstrated encouraging intracranial antitumor activity. Among 21 patients with measurable brain metastases, 11 demonstrated tumor shrinkage in the target brain lesion at AZD3759 doses of ≥50 mg BID. In this group, there were six partial responses (three confirmed, three unconfirmed). Among 22 patients with measurable extracranial lesions, eight experienced tumor shrinkage, with one unconfirmed partial response (60). Based on these promising findings, the BLOOM trial is continuing to enroll patients in the AZD3759 brain and leptomeningeal metastasis expansion cohorts.

## Mechanisms of Resistance to Third-Generation *EGFR* TKIs

As is the case with first and second-generation *EGFR* TKIs, mutations mediating resistance to third-generation *EGFR* TKIs are emerging (61–65). Among them, while the C797S mutation in exon 20 of *EGFR* was the most common mechanism responsible for resistance to osimertinib (62), it occurs in less than 3% of patients treated with rociletinib (66). The C797S mutation was also reported in one case that led to resistance to olmutinib (65). Very recently, two novel tertiary *EGFR* mutations were described. The acquired L798I mutation was observed in *cis* with T790M in one patient following rociletinib therapy (66). Subsequently, another mutation in the same codon (L798Q) was reported in one patient at the time of progression under osimertinib (67).

The acquired resistance associated with the *EGFR* T790M mutation can occur by selection of preexisting *EGFR* T790M+ clones or *via* genetic evolution of initially *EGFR* T790M− drug-tolerant cells, suggesting that cancer cells that survive third-generation TKIs may serve as a key reservoir from which acquired resistance can emerge during treatment (68).

Additional *EGFR*-independent mechanisms of resistance have been reported. *NRAS* mutations, including a novel E63K mutation, and amplifications of wild-type *NRAS* or *KRAS* have been described as mechanisms of acquired resistance to osimertinib but also to gefitinib and afatinib (69). Amplifications in *HER2* and *MET* genes were also described as potential mechanisms of acquired resistance to osimertinib and rociletinib in *EGFR* T790M+ NSCLC patients (66, 70). Additionally, loss of T790M at the time of progression may be mediated by overgrowth of cells harboring *HER2* amplification, or *BRAF* V600E or *PIK3CA* mutations, as was recently detected in plasma of patients included in the phase I AURA trial (71).

Finally, small-cell lung cancer transformation was seen in two cases of rociletinib resistance and one osimertinib-resistant patient; the T790M was lost while the original *EGFR* mutation was maintained in the small cell transformed cancer in each case (72, 73).

## Overcoming Resistance to Third-Generation *EGFR* TKIs

The favorable toxicity profiles of the third-generation *EGFR* TKIs make them particularly attractive candidates for combination therapy, and many trials are currently planned or ongoing (Table 3).

**Table 3:** **Ongoing and forthcoming third-generation *EGFR* TKIs-based combination trials**.

Third-generation *EGFR* TKI	Trial, NCT number	Drug combination	Mechanism of action	Population and setting	Primary endpoint	Status
Osimertinib	NCT02143466; TATTON Phase Ib	Durvalumab Savolitinib Selumetinib	Anti-PD-L1 antibody *MET* inhibitor *MEK* inhibitor	Advanced *EGFR*-mutant NSCLC progressing under *EGFR* TKI	Part A: safety and tolerability Part B: safety, tolerability and efficacy	On hold Recruiting Recruiting

Osimertinib	NCT02454933; CAURAL Phase III	Osimertinib monotherapy		*EGFR* mutant/T790M+ NSCLC progressing under *EGFR* TKI	PFS	On hold
		Durvalumab	Anti-PD-L1 antibody			

Osimertinib	NCT02496663; phase I	Necitumumab	Anti-*EGFR* antibody	Advanced *EGFR*-mutant NSCLC progressing under *EGFR* TKI	Safety and tolerability	Recruiting

Osimertinib	NCT02803203; phase I/II	Bevacizumab	Anti-*VEGF* antibody	Advanced *EGFR*-mutant NSCLC in the first-line setting	Phase I: MTD Phase II: PFS	Recruiting

Osimertinib	NCT02789345; phase I	Necitumumab Ramucirumab	Anti-*EGFR* antibody Anti-vascular endothelial growth factor receptor 2 antibody	*EGFR* mutant/T790M+ NSCLC progressing under first-line *EGFR* TKI	ORR	Forthcoming

		Necitumumab + ramucirumab				

Osimertinib	NCT02520778; phase Ib	Navitoclax	Bcl-2 family inhibitor	Advanced *EGFR*-mutant NSCLC progressing under *EGFR* TKI	Safety and tolerability	Recruiting

Osimertinib	NCT02503722; phase I/II	Sapanisertib	TOR1/2 inhibitor	Advanced *EGFR*-mutant NSCLC progressing under *EGFR* TKI	Safety and recommended phase II dose Safety and efficacy in T790M− population	Recruiting

Nazartinib	NCT02335944; phase Ib/II	INC280	*MET* inhibitor	*Ph. Ib/Ph. II Group 1*: advanced *EGFR*-mutant NSCLC progressing under G/E/A *Ph. II Group 2*: advanced NSCLC not been previously treated with any *EGFR* TKI and harbor *de novo* T790M mutation	Phase Ib: MTD or RP2D of nazartinib Phase II: ORR	Recruiting

Nazartinib	NCT02323126; phase II	Nivolumab	Anti-PD-1 antibody	*EGFR* mutant/T790M+ NSCLC progressing under first-line *EGFR* TKI	PFS	On hold

*G/E/A, gefitinib/erlotinib/afatinib; MTD, maximum tolerated dose; NCT number, http://clinicaltrials.gov identification number; NSCLC, non-small-cell lung cancer; ORR, objective response rate; PFS, progression-free survival; RP2D, recommended phase 2 dose*.

Preclinical *EGFR* L858R/T790M/C797S mutation cell models exhibited *in vitro* sensitivity to cetuximab, an antibody that blocks *EGFR* dimerization (74, 75), but this was not confirmed in *in vivo* analyses. However, the allosteric inhibitor EAI045 in combination with cetuximab exhibited mechanistic synergy and was effective in mouse models of lung cancer driven by *EGFR* L858R/T790M and by *EGFR* L858R/T790M/C797S (76). Interestingly, the allelic context in which C797S was acquired may predict responsiveness to subsequent TKI treatments. For example, if the C797S and T790M mutations are in *trans*, cells will be resistant to third-generation *EGFR* TKIs but are sensitive to a combination of first and third-generation TKIs, and when C797S develops in T790 wild-type cells, this results in resistance to third-generation TKIs, while sensitivity to first-generation TKIs is retained (61). These data are of great clinical value in sequencing for this mutation in patients with acquired resistance to osimertinib.

Navitoclax (ABT-263), a BCL-2 family inhibitor, enhances the apoptotic response of late-resistant *EGFR* T790M cells with decreased sensitivity to *EGFR* inhibition. The combination of navitoclax with the third-generation *EGFR* TKI WZ4002 (in preclinical development) induced more apoptosis compared to WZ4002 alone in both *in vivo* and *in vitro* analyses. This approach could be an effective strategy for treating *EGFR* T790M-positive cancers that have a decreased apoptotic response to *EGFR* inhibition (68). Additionally, the combination of WZ4002 with trametinib, another *MEK* inhibitor, prevents the development of acquired resistance in *EGFR*-mutant lung cancer models (77). A phase Ib trial is ongoing to evaluate the safety and tolerability of the osimertinib/navitoclax combination in patients with *EGFR*-mutant NSCLC following resistance to prior *EGFR* TKIs (NCT02520778).

*In vitro*, a combination of osimertinib with the *MEK* 1/2 inhibitor selumetinib prevented emergence of resistance in PC9 (Ex19del) cells and delayed resistance in NCI-H1975 (L858R/T790M) cells. *In vivo*, concomitant osimertinib with selumetinib caused regression of osimertinib-resistant tumors in an *EGFR*-mutant/T790M transgenic model (69). This association, among others, is being evaluated in the phase Ib TATTON trial (NCT02143466) designed to evaluate the safety, tolerability, and preliminary antitumor activity of osimertinib in combination with durvalumab (an anti-PD-L1 monoclonal antibody), savolitinib (*MET* inhibitor) or selumetinib in patients with advanced *EGFR*-mutant NSCLC who have progressed on an *EGFR* TKI. Preliminary results from the osimertinib/durvalumab arm were recently presented (78). The investigator-assessed ORR was 67% in nine patients with T790M+ tumors, compared to 21% in 14 T790M− NSCLC. Interstitial lung disease was reported in 38% (13/34) of patients, which is higher than would be expected with either drug alone, including five grade 3/4 events (78). Thus, recruitment in the osimertinib + durvalumab arm was stopped but expansion cohorts of the *MET* and *MEK* inhibitor combinations are ongoing. In addition, the phase III CAURAL trial (NCT02454933) is being conducted in second-line metastatic *EGFR*-mutant T790M+ NSCLC patients testing osimertinib plus durvalumab vs. osimertinib monotherapy. This study was also stopped prematurely due to the pulmonary toxicity observed in the TATTON trial.

Dual *EGFR* blockage is being evaluated in a phase I trial (NCT02496663) combining osimertinib with the anti-*EGFR* monoclonal antibody necitumumab to assess safety and determine the optimal dose in patients with *EGFR*-mutant advanced NSCLC who have progressed on a previous *EGFR* TKI.

As was reported, the dual vascular endothelial growth factor receptor (VEGFR) and *EGFR* blockade inhibit tumor growth in *EGFR* TKI resistance xenograft models (79). This hypothesis was confirmed in two phase II clinical trials in *EGFR*-mutant NSCLC treatment-naïve patients, the randomized Japanese (JO25567) trial comparing erlotinib plus bevacizumab vs. erlotinib alone, and the single-arm Caucasian (BELIEF) trial. Median PFS was similar and encouraging in both trials supporting the combination in the first-line setting (80, 81). Following this strategy, a phase I trial was designed to evaluate the safety of two osimertinib-based combination strategies, with necitumumab or ramucirumab (an anti-*VEGFR2* monoclonal antibody) in patients with advanced *EGFR* T790M+ NSCLC after progression on first-line *EGFR* TKI therapy (NCT02789345). Finally, the osimertinib/bevacizumab combination will be evaluated in another phase I/II 3 + 3 dose-escalation study (NCT02803203) to test the safety of combining these drugs.

For patients whose tumors undergo small-cell lung cancer transformation, platinum-based plus etoposide chemotherapy is recommended.

## Conclusion

Over the last decade, we have seen considerable advances in the treatment of patients with *EGFR* mutant NSCLC. Three *EGFR* TKIs are currently FDA and EMA approved for first-line treatment of patients with sensitizing *EGFR* mutations in metastatic NSCLC. Despite this progress, the development of acquired resistance is an unfortunate reality and remains an important challenge in the clinical setting. No second-generation TKIs have been successfully developed, and to date, osimertinib is the only third-generation *EGFR* mutant/T790M+ TKI approved by the FDA and EMA for patients with advanced T790M NSCLC who progress on a first-line *EGFR* TKI. Osimertinib has demonstrated strong efficacy and safety data in phase I and II studies, mainly in a second- or post-second-line setting but also as first-line treatment, placing it as a very attractive drug in this scenario. The clinical development of osimertinib represents one of the fastest cancer drug development programs, taking just 2 years, 8 months, and 1 week from the first patient dosed to the first approved indication. Until recently, patients with advanced NSCLC with *EGFR*-activating mutations who progress on a first-line *EGFR* TKI have traditionally been treated with a platinum-doublet chemotherapy. These combinations show ORRs of approximately 30%, marginally higher than those observed in the T790M− populations, but significantly lower than those reported in T790M+ cohorts across osimertinib phase I–III trial development. In addition, given the encouraging CNS efficacy, osimertinib is also attractive as frontline treatment for patients with brain and/or leptomeningeal metastases. The phase III FLAURA (NCT02296125) trial will hopefully soon answer the issue of where osimertinib should be positioned. Among the other new-generation *EGFR* TKIs and considering that the development of rociletinib and olmutinib as monotherapies has been stopped, ASP8273 is now the most advanced agent in the clinic.

**Figure 2:**
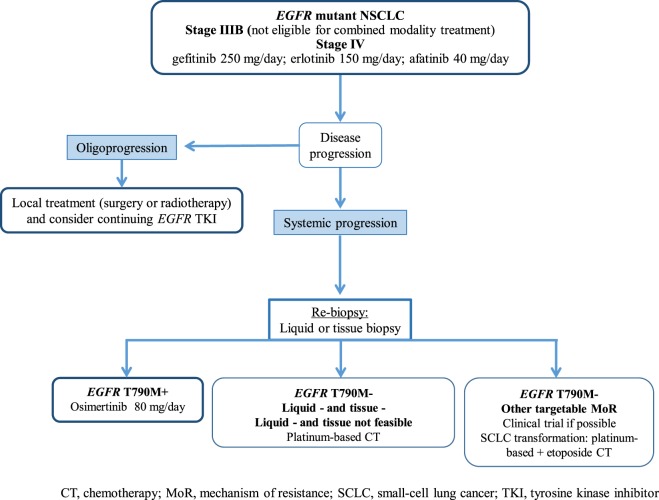
**Potential treatment algorithm for advanced *EGFR*-mutated NSCLC patients**. CT, chemotheraphy; *EGFR*, epidermal growth factor receptor; MoR, mechanism of resistance; NSCLC, non-small-cell lung cancer; SCLC, small-cell lung cancer; TKI, tyrosine kinase inhibitor.

Figure 2 illustrates potential post-progression treatment algorithms for *EGFR*-mutated advanced NSCLC patients. The heterogeneity of resistant cancers seems to play an important role in both efficacy and resistance to these novel T790M-specific agents, and combination strategies could be effective in delaying and/or preventing resistance. Finally, in an era of personalized medicine, the analysis of both tumor tissue and ctDNA should be a priority to improve our knowledge to the benefit of our patients.

## Author Contributions

IS prepared the manuscript. DP supervised and accepted the final version.

## Conflict of Interest Statement

IS reports no conflicts of interest. DP: Advisory Boards for AstraZeneca, Pfizer, Novartis, Clovis, and Roche.
